# Polar Microalgae: New Approaches towards Understanding Adaptations to an Extreme and Changing Environment

**DOI:** 10.3390/biology3010056

**Published:** 2014-01-28

**Authors:** Barbara R. Lyon, Thomas Mock

**Affiliations:** School of Environmental Sciences, University of East Anglia, Norwich Research Park, Norwich NR4 7TJ, UK; E-Mail: B.Lyon@uea.ac.uk

**Keywords:** polar microalgae, physiology, genomics, proteomics, biogeochemistry, sea ice, oceanography, adaptation, evolution, environment

## Abstract

Polar Regions are unique and highly prolific ecosystems characterized by extreme environmental gradients. Photosynthetic autotrophs, the base of the food web, have had to adapt physiological mechanisms to maintain growth, reproduction and metabolic activity despite environmental conditions that would shut-down cellular processes in most organisms. High latitudes are characterized by temperatures below the freezing point, complete darkness in winter and continuous light and high UV in the summer. Additionally, sea-ice, an ecological niche exploited by microbes during the long winter seasons when the ocean and land freezes over, is characterized by large salinity fluctuations, limited gas exchange, and highly oxic conditions. The last decade has been an exciting period of insights into the molecular mechanisms behind adaptation of microalgae to the cryosphere facilitated by the advancement of new scientific tools, particularly “omics” techniques. We review recent insights derived from genomics, transcriptomics, and proteomics studies. Genes, proteins and pathways identified from these highly adaptable polar microbes have far-reaching biotechnological applications. Furthermore, they may provide insights into life outside this planet, as well as glimpses into the past. High latitude regions also have disproportionately large inputs into global biogeochemical cycles and are the region most sensitive to climate change.

## 1. Introduction

Low-temperature environments represent probably the largest untouched biological resource on our planet because the largest proportion of the Earth’s biomass exists in low temperate environments, largely marine. Polar microalgae, which form the base of a largely bottom-up controlled polar food web [1] have successfully adapted to the extreme and oscillating polar environmental gradients. In addition to freezing temperatures, these cold environments coincide with a host of other environmental challenges including solar, osmotic, oxidative and nutrient stress which have been well described in previous reviews [2,3,4]. The ephemeral nature of one of polar microalgae’s major niches, sea-ice, makes it one of the most dynamic of the extreme environments on earth. The semi-enclosed sea-ice habitat harbours a very diverse community of organisms interacting on a very small scale, continually acclimating and adapting to strong and oscillating environmental conditions [5]. This promotes fast evolution through horizontal exchange and recombination of genetic material. Thus, these organisms represent a resource for identification of new species, new physiological mechanisms of adaptation and new genes. However, global warming due to increased atmospheric carbon dioxide concentrations has begun to seriously threaten the coldest environments on our planet, polar ecosystems. This could mean a loss of a vast pool of genetic diversity yet to be uncovered. And only through advances in our understanding of molecular mechanisms driving metabolism and community structure of polar microalgae will scientists be able to better estimate the current and future biogeochemical inputs and niche adaptability of polar microalgae.

The focus of this review is to describe the physiological mechanisms involved in microalgae adaptations to cryospheric conditions, emphasizing insights “omics” techniques have recently provided. The later section, “Using systems biology to understand a changing world,” highlights gaps in knowledge and suggested priorities for future research with particular emphasis on “omics” applications. We also refer readers to previous reviews that have given insights into microalgae adaptations to polar environments, including several which have gone into great detail on sea-ice structure, biodiversity, primary production and niche adaptation [5,6,7], and those that have focused on polar lake microalgae [8,9] and cyanobacteria [10,11], as well as reviews of polar macroalgae [12] and bacteria [13,14,15,16,17]. There have also been very informative reviews of the metabolic and biogeochemical insights derived from the first two temperate microalgae genomes sequenced [18,19]. More general reviews of metagenomic [20], proteomic [21], and metabolomic [22] environmental applications are also available.

## 2. Polar Significance

Polar regions are unique, prolific ecosystems despite their inhospitable appearance [23]. The Antarctic continent covers an area of 14 million km^2^ and amidst the world’s largest desert are numerous perennial frozen lakes. It is surrounded by the Southern Ocean, a high nutrient low chlorophyll (HNLC) region, which is covered by seasonal sea-ice that can extend up to 20 million km^2^, covering ~40% of the Southern Ocean during austral winters [24]. Southern polar glaciation arose ~40 million years ago coinciding with the separation of the Antarctic and South American continents and formation of the Antarctic Circumpolar Current [25]. In the northern hemisphere, regions of North America, Europe and Asia continents all lie within the Arctic Circle (66°33'N) and surround a shallow Arctic basin covered by up to 16 million km^2^ of perennial and seasonal sea-ice [26]. However, the most recent northern glaciation event did not occur until ~3.5 million years ago, making this a much younger cryospheric region [25].

Together, these polar regions account for a large proportion of the Earth’s surface area and have great impacts on global biogeochemical cycles. Due to increased CO_2_ solubility at low water temperatures, deep water formation in the Southern Ocean sequesters large amounts of carbon (*i.e.*, 30% of global uptake despite accounting for only 10% of the surface area; (i.e., 30% of global uptake despite accounting for only 10% of the surface area; [27]). High river inputs and the largely refractory nature of the terrestrial dissolved organic matter entering the Arctic basin also leads to large CO_2_ sequestration [28]. However, warming Arctic temperatures are mobilizing frozen methane deposits, a greenhouse gas that can further exacerbate climate warming [29]. Polar regions have also been shown to produce large biogenic sulfur fluxes to the atmosphere through the breakdown of the phytoplankton metabolite dimethylsulfoniopropionate (DMSP) to dimethylsulfide (DMS), a volatile gas [30]. Oxidized sulfate particles from DMS, in turn, help seed clouds which mitigate climate warming [31]. The qualitative and quantitative inputs polar ecosystems have on the various biogeochemical cycles and food webs are largely dependent on the microbial populations that control primary production and remineralization processes. While perennial permafrost severely limits terrestrial photoautotrophs, algae and cyanobacteria have successfully adapted to polar marine and/or fresh water niches. They form the base of these productive food webs, converting light energy and nutrients into chemical energy despite a physiologically challenging environment.

## 3. Microalgal Mechanisms to Thrive

### 3.1. Membrane Fluidity

Cell membranes control transport of nutrients and metabolic waste products in and out of the cells and are integral to the electron transport chains of cellular metabolism. Therefore, maintaining their fluidity under freezing temperatures is of utmost importance. Increases in unsaturated bonds promote a looser packing of lipids and decreased temperature of solidification. Increased concentrations of polyunsaturated fatty acids (PUFAs) is one of the most well-documented cold tolerance mechanisms and has been shown in polar diatoms [32], dinoflagellates [33], and chlorophytes [34,35,36]. Polar microalgae not only increase PUFA concentrations in cell membrane phospholipids, but perhaps more importantly, in the galactolipids integral to the chloroplast membrane [37,38,39,40]. The recent publication of the genome of a psychrotolerant green algae, Coccomyxa subellipsoidea, found amongst the most enriched gene families FA synthases, elongases, lipases, and desaturases [41], highlighting the importance of lipid metabolism under polar conditions. Desaturase enzymes are responsible for inserting double bonds into FAs at specific carbon locations and differential regulation of desaturases indicates locations of double bonds are tightly controlled [42,43]. Upregulation in response to salt stress indicates they are also likely involved in more than just temperature acclimation [44,45]. Unlike bacterial desaturases, de novo PUFA synthesis by eukaryotic desaturases can function independent of growth, which is important for rapid acclimation [9]. However, the sensory and signal pathways involved in PUFA synthesis remain to be elucidated in eukaryotic phytoplankton.

### 3.2. Enzyme Kinetics

Microbes are poikilothermic (*i.e.*, they are in thermal equilibrium with their surrounding environment). Thus, bioenergetic demands of the cell must overcome the inhibiting effects of a low kinetic environment, most notably the freezing of molecules and decreased rates of catalysis. Recently, thanks to the sequencing of >30 prokaryotic genomes, specific protein structural changes promoting cryospheric enzyme flexibility were identified and statistically validated [14]. They include amino acid substitutions which decrease hydrophobic interactions, H-bonds and salt-bridges, particularly around the active site, which in turn can increase reaction rates by requiring less energy than induced fit mechanisms. A detailed review of enzymes kinetics in polar prokaryotes is given by Gerday and colleagues in this issue [15]. While the availability of only two polar microalgae genomes does not enable statistical investigations into amino acid substitutions, physiological and molecular techniques have shown the temperature hardiness of polar microalgae metabolic enzymes utilizes various mechanisms depending on the enzyme. For example, studies of ice diatoms found enzymes involved in nitrate, ammonium and carbon uptake have optimal temperature ranges at near freezing temperatures, while other metabolic enzymes such as nitrate reductase (NR) have more moderate optimal temperatures but less sensitivity to temperature changes [46]. Comparisons between psychrophilic and mesophilic chlorophytes also showed shifts to higher and more stable activity of psychrophilic metabolic enzymes at low temperatures [47,48]. Interestingly, NR from a psychrophilic chlorophyte was capable of utilizing both NADPH and NADH as energetic reductants, whereas the mesophilic chlorophyte could only use NADH [47]. Thus, in response to polar conditions which can reduce metabolic production of energy units, enzymes from polar microalgae seem to have evolved to be more flexible in their energy source. This is supported by the fact that measurements of NR:NADH activity showed maximal activities at higher temperatures than *in vivo* assays; Ferrara and colleagues [49] suggest this can be explained by the *in vivo* presence of enzymes such as glucose 6-phosphate dehydrogenase whose synthesize of NADPH improves other enzymes (*i.e.*, NR) cold activity. Another example of polar microalgae kinetic adaptations was the elevation of pyrophosphate-dependent phospho-fructo-kinase detected in the polar diatom *Fragilariopsis cylindrus* during salinity acclimation [50]. This ATP-independent form of an important glycolysis enzyme saves chemical energy units for other processes, such as osmolyte synthesis. A gene encoding a rhodopsin has also been found in *F. cylindrus* which has been suggested to serve as a trace-metal independent method for additional ATP synthesis [51,52]. Surprisingly, ribulose-1,6-bisphosphate carboxylase (RUBISCO), which is fundamental to phototrophic carbon fixation, shows an opposite trend of greater decreases in activity under cold temperatures when psychrophilic chlorophytes where compared to their temperate counterparts [53]. However, the cellular concentrations of RUBISCO enzymes in the polar species were twice that found in temperate algae [53]. A similar mechanism was observed in molecular studies in *Chlamydomonas subcaudata* which showed increased concentrations of ATP synthase proteins within the chloroplast [54]. Elevations in this enzyme’s abundance may partly explain the ability for psychrophiles, but not mesophiles, to increase ATP production following cold-shock [55]. Most recently ribosomal proteins have also been found to be significantly increased at colder temperatures, presumable to maintain adequate translation within a low kinetics environment [56].

### 3.3. Compatible Solutes and Cryoprotectants

**Figure 1:**
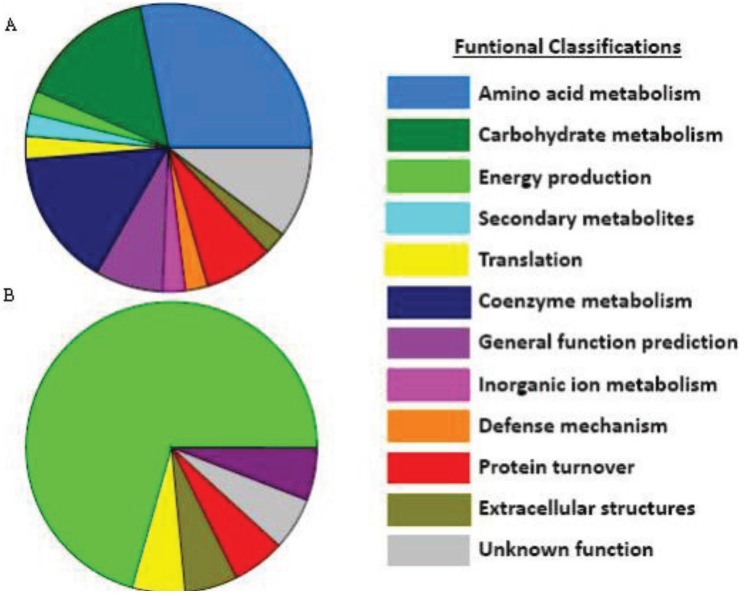
Functional groups for elevated (**A**) and reduced (**B**) proteins in *F. cylindrus* following a shift to high salinity. This is an example of the major functional groups associated with polar algae acclimation processes. Note that most proteins resolved on 2DE gels had predicted functions in contrast to the much larger percentage of unknown proteins in genomic and transcriptomic datasets, most likely due to gel bias towards highly abundant metabolic proteins.

Cellular compatible solutes, typically associated with their role in salinity acclimation, also serve as freeze protection molecules and contribute to the high *in vivo* enzyme activities within psychrophilic organisms. These compounds reduce the intracellular freezing point and help maintain enzyme hydration spheres to stabilize catalytic activity [57]. There is a wide array of compatible solutes including: sugars, polyols, amino acids and their derivatives such as betaine and DMSP. The amino acid proline is an abundant compatible solute in many cryophilic microalgae, including the model ice diatom *F. cylindrus*. Genes for proline synthesis were strongly represented in *F. cylindrus* cold and salt stress EST libraries [58]. Proteomics studies further validated the importance of proline synthesis within this ice diatom with seven of the 36 proteins that increased in relative abundance in response to high salinity involved in the amino acid synthesis pathway of proline ([50]; Figure 1). Two isoforms of a homologue to the bacterial/archaeal glycine betaine methyltransferase protein were also elevated. This is an alternate enzyme for synthesis of the compatible solute betaine that, in contrast to the more typical eukaryotic choline oxidase pathway, produces large betaine concentrations without damaging H_2_O_2_ as a by-product [59]. High concentrations of DMSP, a compatible solute with important feedbacks in climate and biogeochemical cycles, are found in ice-diatom communities [60] in contrast to typically low levels in temperate diatoms [61]. This compound has been shown to stabilize enzymes against cold-induced denaturation [62]. Proteomics following the salinity shift of *F. cylindrus* identified candidate genes for all four steps of the proposed synthesis pathway (from previous radiolabeling metabolite studies by [63]) demonstrating the utility of this “omics” technique to identify candidate enzymes for poorly characterized metabolic pathways [50].

Late embryogenesis abundant (LEA) proteins are also considered multi-faceted cryoprotectants believed to work through hydrogen-bond stabilizing effects on enzymes [64]. LEA genes were found to be highly expressed in clone libraries from the polar chlorophyte *Chlorella vulgaris* (which was recently reclassified as *C. subellipsoidea*) and associated with cold-hardiness [65]. Additional novel LEA proteins were later isolated from this species by suppression subtractive hybridization and shown to protect lactate dehydrogenase activity from freeze inactivation [66]. 

A category of “cold-shock response” proteins has also been identified and is present across all taxa, from microbes to mammals [14]. These encompass a family of highly conserved small molecular weight proteins which bind to cold shock domains of single-stranded nucleic acids, as well as a number of RNA helicases. Cold-shock proteins are highly abundant in *F. cylindrus* two-dimensional protein gels (equivalent intensity to the highly abundant light harvesting complex proteins) and were significantly elevated following shifts from 4 °C to 0 °C [67]. Furthermore, six DNA/RNA helicases were identified within the *F. cylindrus* cold stress EST library [68]. It is believed that they work together to promote replication, transcription and translation under low temperature conditions by minimizing cold denaturation which otherwise forms kinks, coils and secondary structures that impede such processes.

### 3.4. Extracellular Compounds

Under freezing conditions it is also important for phytoplankton to maintain an aqueous external environment. To this end various extracellular modifiers are produced by microalgae within the ice such as ice-binding proteins (IBPs) which are excreted from the cells and inhibit ice growth and recrystallization and enhance brine retention through changes in ice channel structure [69]. IBPs were first identified in ice diatoms simultaneously in the salt shock EST library of *F. cylindrus* and through protein isolation and mass spectrometry from the ice-diatom *Navicula glaciei* [70]. The presence of these genes in all ice-algae tested to date (diatoms, prymnesiophytes, prasinophytes and chlorophytes), and complete absence from temperate species, suggests that IBPs play an important role in sea-ice adaptation [71]. Furthermore, phylogenetic analysis strongly suggests the genes were acquired through horizontal gene transfer (HGT) of bacterial IBPs [71,72]. Exopolymeric substances (EPS), composed of polysaccharides, amino acids, and proteins, are also highly abundant within the sea-ice [73]. Diatom EPS also helps retain salts, increase liquid brine fraction and thus create microhabitats within sea-ice [74]. Polysaccharide and cell wall metabolism gene families, which likely includes enzymes for synthesis of EPS and antifreeze glycoproteins, were enriched in the polar green algae *C. subellipsoidea* compared to temperate chlorophytes [41]. The role of EPS in polar adaptation is nicely reviewed by Ewert and Deming [7] within this special issue.

### 3.5. Light Acclimation

Light availability is highly variable in the polar environment and polar microalgae must both avoid photodamage under periods of high light and adapt to low light levels. Ice-diatoms are well adapted to low light levels. High photosynthetic efficiencies enable them to reach saturated growth at 20 µE m^−2^ s^−1^ and active photosynthesis has been observed at irradiances < 0.5 µE m^−2^ s^−1^, 0.01% of incident irradiance [75,76]. Physiological studies revealed steady-state *F. cylindrus* cultures growing at 2 µE m^−2^ s^−1^ *versus* those grown at 15 µE m^−2^ s^−1^ exhibited increases in specific chloroplast PUFAs that enhance the fluidity of the thylakoid membrane and thus the flow of electrons, and this was associated with a near doubling of pigment concentrations and 50% reduction in carbohydrate concentration [38]. Fucoxanthin-chlorophyll binding proteins (FCPs), complex families of proteins that appear to have different specializations (e.g., light harvesting *versus* dissipation of excess energy), were the most redundant genes identified in *F. cylindrus* and *C. neogracile* EST libraries [68,77] but it has yet to be tested whether elevated FCP concentrations are also part of the high shade adaptability of polar diatoms. However, it appears that in ice diatoms a dense packaging of pigments and their binding proteins in conjunction with enhanced thylakoid fluidity enable high photosynthetic efficiencies at very low light, while carbohydrate utilization and alternate energy sources help offset any energetic deficiencies. The chlorophyte *Chlamydomonas raudensis*, which dominates the highly saline bottom layers of permanently ice-covered Antarctic lakes, is also highly shade adapted, but utilizes quite unique mechanisms (reviewed in [9]). It has lost many conserved short-term and long-term photoacclimation mechanisms such as non-photochemical quenching (NPQ), light harvesting complex state transitions between PSI and PSII, and alterations in pigment concentrations. Instead, *C. raudensis* has an extremely high PSII to PSI stoichiometry to maximize harvesting of low levels of blue light. Furthermore, in response to increased irradiance (up to 10-fold higher than natural conditions) *C. raudensis* increases growth rates to dissipate increased energy rather than exhibiting photoinhibition. In contrast, the chlorophyte species (Chlorella BI) isolated from an Antarctic pond, with higher and more fluctuating light levels, has maintained its ability for state transitions to balance PSI and PSII light absorption and thus maintain optimal photosynthetic activity under changing light conditions [78]

Despite their adaption to low light levels, ice diatoms are still capable of acclimating to high light (>350 µE m^−2^ s^−1^) at temperatures down to −5 °C [79]. Like diatoms from other habitats, they utilize NPQ mechanisms, such as the diatoxanthin - diadinoxanthin xanthophyll cycle, to dissipate excess energy and prevent photoinhibition and cellular damage [80]. Xanthophyll cycle pigments can bind to the LHCx family of FCPs associated with the dissipation of excess energy [81]. The genome of *F. cylindrus* revealed a large expansion in the LHCx gene family compared to the two sequenced temperate diatom species [82]. Microarray studies with the polar diatom *Chaetoceros neogracile* found shifts from 20 to 600 µE m^−2^ s^−1^ resulted in significant elevations in LHCx proteins and antioxidant proteins, while those associated with light harvesting were significantly reduced [83]. The fact that growth rate was only 20-35% reduced over a 10 day period at this very high light level illustrates the photoacclimation capabilities of this species. Interestingly, work from various labs has shown low temperatures to elicit photoacclimation responses similar to high light, such as increased NPQ, PSII proteins, and photoprotective pigments [35,79,84]. Presumably these changes were a result of increased excitation pressures caused by low temperatures (*i.e.*, decreased enzyme kinetics inhibits efficiency of metabolic electron sinks leading to a build-up of reduced plastoquinone). The redox status of the plastoquinone pool in turn triggers phosphorylation cascades which initiate photoacclimation mechanisms [81,85]. Salinity shifts can also elicit photoacclimation mechanisms in polar chlorophytes and diatoms [67,86], again likely through the common mechanism of changes in excitation pressures. Thus, multiple environmental pressures require robust photoacclimation mechanisms for microalgae to thrive within the cryosphere.

### 3.6. Antioxidants

Photosynthesis creates an oxic environment intracellularly that can be exacerbated by reactive oxygen species (ROS) formation caused by low temperature and other stress induced metabolic imbalances. This is further magnified by the increased solubility of oxygen at low temperatures and restricted diffusion within sea-ice and permanently ice-covered lakes leading to hyperoxic extracellular environments. Thus, robust antioxidant systems are important for polar microalgae to cope with ROS. Polar diatoms [87], chlorophytes [88] and dinoflagellates [89] are resistant to UV damage, indicating highly effective antioxidant systems. High catalase activity in the sea-ice diatom *Entomoneis kufferathii* following exposure to high light and low temperatures [90] is one of many antioxidant systems that can help protect cells from oxidative damage. Studies with the polar diatom *Chaetoceros brevis* showed elevated levels in superoxide dismutase activity, in addition to xanthophyll cycling, also to be important for dissipating ROS brought on by irradiance shifts [91]. A survey of antioxidant systems showed polar algae also utilize ascorbate peroxidase and glutathione reductase as antioxidant systems; however, the activity levels following light stress did not show a clear geographic trend between polar and temperate species but rather indicated species specific responses that could be due to differences in photoacclimation capabilities and utilization of alternate antioxidant systems [92]. In fact a microarray study with the polar diatom *Chaetoceros neogracile* found amongst thermal stress response genes (following shift from 4 °C to 10 °C) a spectrum of antioxidant enzymes, including monoascorbate reductase, glutaredoxin, glutathione peroxidase, glutathione S-transferase (GST), and alternative oxidase, illustrating the diverse suite of ROS defense enzymes this psychrophile can utilize to mitigate oxidative damage [93]. Various compatible solutes such as proline and DMSP also have secondary benefits as antioxidants [94,95] and their high concentrations in polar microalgae likely contribute to protection from oxidative damage. Furthermore, symbiotic relationships between ice-diatom and epiphytic bacteria who scavenge ROS have been described [96].

Given the importance of PUFAs to cryoprotection and their sensitivity to oxidation, protection against ROS damage to these lipids is of utmost importance. GST conjugates reduced glutathione to electrophilic centers, particularly abundant within PUFAs, and protects them from more damaging oxidation such as lipid peroxidation and other reactions with H_2_O_2_. In addition to the GST transcript elevations in response to elevated temperatures mentioned above, proteomics studies have found significantly elevated GST protein levels to be involved in low temperature acclimation of the sea-ice chlorophyte, *Chlamydomonas sp.* [97] and high salinity acclimation of the polar diatom, *F. cylindrus* [50], further emphasizing its importance in environmental stress tolerance across diverse taxa of polar microalgae. Another important enzyme for protecting cellular proteins from oxidative damage is methionine sulfoxide reductase (Msr) which reduces methionine residues that become oxidized by ROS. MsrB has been shown to be a cold response protein in the *Arabidopsis* plant and knock-down mutants show decreased cold tolerance in the form of increased methionine oxidation, H_2_O_2_ formation, and electrolyte leakage [98]. Fourteen Msr homologues are present in the *F. cylindrus* genome (*versus* seven and ten in the temperate diatom genomes of *Thalassiosira pseudonana* and *Phaeodactylum tricornutum*, respectively) supporting an important role for Msr enzymes in cold and oxidative stress acclimation of polar microalgae. Comparisons between the genome of the psychrotolerant chlorophyte *C. subellipsoidea* and its temperate counterpart found enrichment in the polar species of a family of short-chain dehydrogenase/reductase enzymes whose substrates vary from alcohols, sugars, and steroids to xenobiotics [41]. Most proteins in this family have oxidoreductase activity and enrichment could indicate an important role in maintaining a balanced cellular redox state under polar conditions, perhaps complementary to aldehyde dehydrogenases mitigation of chemically reactive oxidized lipid aldehydes that can accumulate when environmental stress perturbs metabolic balance as shown in plants [99]. Interestingly, an aldehyde dehydrogenase protein was found to be significantly elevated in *F. cylindrus* during high salinity acclimation at 0 °C [50]. While there is still much work to be done to understand more thoroughly the molecular mechanisms enabling oxidative stress tolerance in polar microalgae, studies thus far indicate a diverse suite of mechanisms to prevent and mitigate ROS damage.

### 3.7. Dark Adaptation

Survival through winter’s extended periods of darkness is key to photoautotrophic success in polar regions. Dark adaptation is also important in controlling microalgae seasonal and spatial distributions [100]. Incubation experiments found temperate diatoms to survive 21–35 days of darkness [100], while Antarctic species survived 4-9 month periods in the dark [101]. However, little is known of physiological mechanisms behind polar overwintering as logistics severely limits austral winter field studies. It is known that carbohydrate storage molecules such as glucan in diatoms and starch in chlorophytes are accumulated within polar algae and utilized during periods of darkness [9,102]. Furthermore, polar microalgae can also uptake dissolved organic material such as sugars and starches for energetic breakdown [103]. A recent study of polar pelagic algae also described a high plasticity in regards to inorganic carbon uptake in Southern Ocean phytoplankton [104]. Comparison of the recent polar chlorophyte genome *C. subellipsoidea* to its temperate counterparts showed gene enrichment in amino acid transporters and permeases which would promote enhanced uptake of organic nutrient sources [41]. Additionally, a number of carbohydrate metabolism gene families were present in *C. subellipsoidea* that did not have homologs in temperate chlorophyte species but instead appeared to have HGT origins. In diatoms, the presence of the urea cycle has been proposed as a means for recovering carbon and nitrogen depleted during photorespiration [18], while the FA β-oxidation pathway means lipids can be used as metabolic intermediates and for ATP synthesis [105]. This metabolic flexibility being revealed through diatom genomes is likely fundamental to their ability to thrive within the extreme and highly-variable environmental conditions which characterize polar regions.

Ice-algae cells can go into a winter resting stage during which time minimal changes in carbon and chlorophyll concentrations occur [106]. Low temperature shift in *F. cylindrus* (5 °C to −1.8 °C) were associated with reductions in photosynthesis and carbon fixation genes, and this was hypothesized to indicate a preparation towards a winter resting stage cued by decreasing temperatures [107]. *Xanthonema*, a class of heterokont snow algae, has been shown to disassemble PSII but keep LHC proteins intact in response to extended dark adaptation [108]. Thus, it appears they maintain thylakoid structural proteins primed to quickly reassembly PS in order to utilize the first short periods of irradiance in austral spring. Extended dark adaption studies in the chlorophyte *Koliella antarctica* showed similar PS changes, but also detected hallmarks of programmed cell death (PCD) in a subpopulation of cells [109]. However, return of cultures to low light after 60 days of darkness showed rapid recovery of growth and photosynthesis, clearly demonstrating viability of cells that did not undergo PCD and raising the question of PCD as an adaptive benefit to unicellular communities. There is still much debate to whether shifts to heterotrophic metabolism also play a role in adaption to extended periods of darkness. Transformation of *P. tricornutum* with a single gene, a glucose transporter, enabled this diatom to switch from photoautotrophic to heterotrophic growth [110], illustrating the ease for such a shift to occur given the high degree of HGT believed to occur within the sea-ice environment. Indeed, the chlorophyte *Chlorella* BI is capable of switching between autotrophic growth and heterotrophic growth, with highest growth rates achieved during mixotrophic growth in light with a glucose carbon source [78]. Many dinoflagellate species are also capable of heterotrophic growth, and shifts to mixotrophic sea-ice communities have been detected from late winter ice cores [111]. However, more research is needed to understand metabolic shifts occurring within species and through changes in community composition during seasonal periods of extended darkness.

## 4. Using Systems Biology to Understand a Changing World

**Figure 2:**
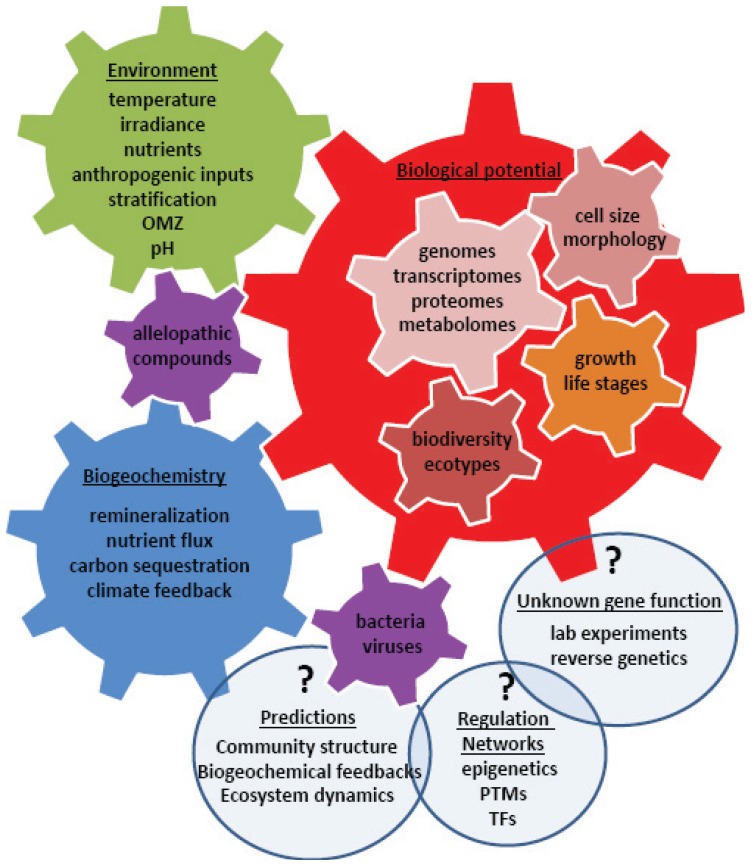
An array of advanced, high-throughput molecular techniques is enabling a global effort to link environmental parameters to biological distributions, physiological capabilities, protein expression, metabolic functions, and biogeochemical cycles. The ultimate goal is the ability to make high resolution predictions of biogeochemical and ecosystem inputs under current and future climates. The light blue bubbles represent areas to focus future research to improve our understanding and modeling efforts. OMZ–oxygen minimum zone, PTM – post-translational modification, TF–transcription factor.

Improving our understanding of the molecular mechanisms behind environmental acclimation and adaptation processes is key to predicting ecological and biogeochemical inputs of polar primary producers (Figure 2). Our molecular tool box has greatly advanced over the past couple decades, and the next hurdle is linking this knowledge to processes on the global scale with ever increasing resolution. While some mechanisms may be unique to polar environments, many are likely utilized in other environments to overcome similar bioenergetic pressures that may arise from common or quite distinct stressors. Temperature, light, nutrients, allelopathic and anthropogenic compounds, and chemical-physical processes (e.g., stratification, oxygen minimum zones, carbonate saturation depth) collectively control temporal and spatial taxonomic distributions depending on the biological potential of organisms (*i.e.*, genetic adaptability). Different evolutionary histories (e.g., endosymbiotic events, HGT, gene loss/expansion during niche specialization) provide different suites of genes which result in metabolic diversity. Differences in metabolism between species, in turn, ultimately result in different impacts on biogeochemical cycles. A systems biology approach to understand the complex genomic, transcriptomic, proteomic, and metabolomic interactions is required if we are to achieve a holistic understanding of feedbacks between organisms and their environment and eventually develop mathematical models capable of representing past, current, and predicting future biological inputs on various ecosystem parameters, such as climate, biodiversity, and nutrient availability (Figure 2). Measuring the effects of various environmental variables, individually and in combinations, on the vast array of phytoplankton is a laborious and logistically difficult matrix of experiments. Alternatively, environmental “omic” approaches reveals the importance of genes in the natural environment and can be correlated with environmental metadata to tease out significant relationships and important drivers of biodiversity, gene expression, and biogeochemical inputs. But in order to move beyond the current low resolution, broad taxonomic group models there are some important gaps (highlighted in the following paragraphs) that must be addressed, particularly with regard to polar primary producers due to their significance in global biogeochemical processes and the sensitivity of this region to climate change.

Polar microalgae genomes provide valuable information on their genetic repertoire, as well as promoter and intergenic regions whose diverse roles in gene regulation we are still just beginning to understand. Polar microalgae genomes also can provide novel proteins and pathways for biotechnological applications and insights into cryosphere bioenergetics that may even help us understand possibilities for life outside of this planet [112]. The *F. cylindrus* genome was the first psychrophilic microalgae genome to become publicly available [113] and insights from its annotation should be published in the near future. Recently the genome of a psychrotolerant green algae, *C. subellipsoidea*, was published [41]. Sequencing of a dominant polar haptophyte, *P. antarctica*, has also been started by the U.S. Department of Energy Joint Genome Institute in 2010 and likely will become public in 2014 (Arrigo, personal communication). Thanks to the rapid advancement of sequencing and annotation techniques, genome sequencing costs and time has been greatly reduced and the number of sequencing facilities has increased substantially. Thus science is now capable of an important next step to understanding polar microalgae physiology and global biogeochemical inputs: expand reference genomes and/or transcriptomes to include multiple species from various taxa representing different niche specialists, and importantly environmental isolates rather than clones that have been cultured for decades in the laboratory. Comparisons between ecotypes of the same species (*i.e.*, cosmopolitan species like *C. neogracile* and *C. raudensis* with polar and warm water strains) will also be incredibly valuable to understanding genes fundamental to cryospheric life. To help fill this gap The Gordon and Betty Moore Foundation has undertaken the Marine Microbial Eukaryote Transcriptome Sequencing Project [114] to sequence the transcriptomes of 750 samples from a diverse range of habitats, including many more polar species. This will enable us to better understand competitive advantages between species and ecotypes and differences in their metabolic potentials. Importantly, it will also substantially increase our database of reference genomes for identifying the taxonomic source of environmental metatranscriptome and metaproteome sequences and prevent misassignment due to limited reference libraries. Furthermore, a new technique of single-cell sequencing can now be used to provide genetic information on non-cultured environmental samples and detect differences and interactions at the individual cell level [115].

Determining which species are present and contributing to biogeochemical fluxes is not a simple task. Since traditional microscopy methods are laborious, molecular techniques have been gaining prominence. Hypervariable regions of the eukaryotic 18s small subunit ribosomal RNA (rRNA) sequence have been used to define eukaryotic phytoplankton taxonomic units at the level of species supergroups (*i.e.*, division or family level) and this has greatly expanded our appreciation of polar microalgae diversity, particularly with regards to the more fragile single-celled organisms [116,117]. Using 18s rRNA Charvet and colleagues [118] found the diversity of Antarctic lake flagellates was significantly underestimated by traditional microscopy and HPLC pigment methods. Similarly, 18s rRNA studies of Antarctic sea-ice communities under different irradiance regimes identified a very diverse eukaryotic community and identified three seasonal stages in community structure: mixed, dinoflagellate-dominated, and diatom-dominated [119]. However, it is important to note that 18s rRNA methods have their own biases towards particular species depending on the primer pairs used [120]. Furthermore, 18s rRNA copy number varies substantially between species [121]. And many 18s rRNA environmental sequences cannot be assigned to known taxa, as evidenced by preliminary analysis of the recent TARA expedition which could not assign 31% of 18s rRNA v9 sequences to known supergroups [122]. So while molecular taxonomic techniques increase sensitivity to species richness and provide a high-throughput means for gathering phenotypic information to link with metatranscriptome and metaproteome data, to apply 18s rRNA metagenomic techniques beyond a presence/absence assessment will require a greatly expanded reference database and increased efforts to quantify and normalize inherent sequencing biases. Furthermore, to truly improve our understanding of who is where and doing what will require the development of molecular markers able to define microalgae taxa with ever increasing specificity, eventually at the species and ecotype level.

Genes of unknown function made up six of the 10 most abundant genes in the *F. cylindrus* EST libraries [68] and 45% of the 1700 unique ESTs in *C. neogracile* [77]. While genes of unknown function are a common feature of all genomes, there are great possibilities for metabolic ingenuity and novel functions amongst these genes in polar microalgae. Environmental expression and functional characterization studies will be key to understanding their effect on an organisms biological potential and biogeochemical feedbacks (Figure 2). Even the assignment of genes to putative functions (*i.e.*, aldehyde dehydrogenase mentioned earlier) are usually only based on sequence similarity and conserved domains that place them in broad enzyme classes with a diverse array of cellular functions, which in a eukaryotic cell are also dependent on subcellular localization. This is further compounded by redundant enzymes and paralogs within a genome. Thus, while comparative “omics” using natural and laboratory controlled conditions provides candidate genes for important and novel regulatory and metabolic processes, a true understanding of function and activity will require protein characterization studies using over-expression and silencing transformations within appropriate host organisms. Such work is easy and well-established in bacterial systems, but these hosts lack eukaryotic organelles, chaperones, post-translational systems and the upstream and downstream signaling networks native to that gene. Gene overexpression and silencing techniques have been developed in temperate diatom species *P. tricornutum* and *T. pseudonana* [110,123,124]. However, the establishment of methods to transform polar diatoms and other phytoplankton taxa would greatly enhance our ability to functionally characterize novel polar genes. New techniques such as viral promoters and transcription activator-like effector nucleases are promising approaches to expand this technology into polar microalgae host models.

Various cell organelles and metabolic systems are involved in polar acclimation processes and interact through a complex network of sensory, signaling, and regulatory mechanisms. Understanding and predicting biogeochemical feedbacks requires untangling and ultimately quantifying this collection of synergistic and antagonistic intracellular interactions. In contrast to the large proportion of putative genes with unknown functions found in *F. cylindrus* transcript studies, only three of the 56 proteins identified in an *F. cylindrus* proteomics study were of unknown function (Figure 1; [50]). Protein gels are biased towards highly abundant and soluble proteins. Perhaps these strongly differentially-regulated gene transcripts of unknown function code for unique signaling and transcription factor proteins which do not need to be expressed at the same concentrations as metabolic enzymes to have immense physiological effects, hence their limited presence in differentially expressed 2D gel proteins. A high priority for understanding gene expression on a systems biology level must be deciphering gene networks, transcription factors and promoter binding regions, sensory/signaling pathways and post-translation regulation. Such studies have been applied to plants and cyanobacteria but are still in their infancy within temperate microalgae and have yet to be applied to polar counterparts.

The ability of polar microalgae to acclimate to a wide range of environmental gradients and their broader range in photosynthetic and metabolic responses compared to temperate counterparts [125] has led to the conclusion that they possess a high degree of phenotypic plasticity. Phenotypic plasticity is like a “cellular memory” that responds to environment and may be selected for in environments with multiple stressors and steep or rapidly changing gradients [19,126]. Epigenetic modifications, such as cytosine methylations and histone modifications can serve as “soft” heritable changes which effect transcription. Recently, a high level of methylation of transposable elements (TEs) and a subset of genes which tended to be involved in important metabolic activities of nutrient resource management were identified in the “methylome” of the diatom *P. tricornutum* [127]. Environmental cues, such as nitrate limitation, decreased methylation of specific genes and triggered an increase in transcript levels. On the other hand, TEs are mobile genetic units and activation by environmental stressors (likely through changes in methylation) can lead to genetic rearrangements, another mechanism facilitating evolution and environmental adaptation at a rapid rate [128]. Small non-coding (silencing) RNAs are another method for controlling phenotypic plasticity in plants and animals [129] and cDNA libraries generated from *T. pseudonana* small RNAs indicate that these are also likely important transcription and translation regulators in microalgae [130]. Clearly, there are still many gaps in our understanding of the molecular mechanisms behind the high level of phenotypic plasticity within polar microalgae which must be addressed if we are to begin quantifying environmental regulation of cellular feedbacks into biogeochemical processes.

Microalgae community structure, genetic mobility, and nutrient availability are all regulated by bacteria and viruses [131] which are abundant in polar environments [132]. Recently a study from an Antarctic hypersaline lake described a virophage-virus-prasinophyte interaction whereby the virophage limited virus-induced mortality and increased phytoplankton blooms [133]. Viruses have also been shown to stimulate PCD pathways in phytoplankton [134]. On the other hand, bacteria-derived infochemicals may stimulate diatom EPS capsule formation [135] and species-specific interaction between a bacterium and diatom may enhance diatom growth, likely through a bacteria produced phytohormone [136]. Yet only a few of the potential bioactive secondary metabolites (aka allelopathic compounds or infochemicals) responsible for microbial intra/interspecies communication have been described so far. These include diatom derived aldehydes shown to trigger nitric oxide signalling and PCD in phytoplankton [137] and diatom derived oxylipins, formed from oxygenated PUFAs and shown to disrupt zooplankton reproduction and development [138]. Studies specific to polar microbial communities are still lacking. The role of intra/interspecies communication and predator-prey interactions in polar ecosystems, particularly in regard to viral and bacterial regulation of primary production, bioremineralization processes, community structure, and biogeochemical cycling, should be a high priority of future research.

For some time now polar regions have shown an amplified sensitivity to climate change [139]. Increased temperatures and winds have increased sea-ice retreat, upper ocean freshening and nutrient upwelling in the relatively shallow Arctic basin; this, in turn, has increased pelagic primary production but with a shift from nano to picoplankton populations [140]. Thinning sea-ice, allowing for increased light penetration, has also led to large under ice phytoplankton blooms in the Arctic [141]. In the Southern Ocean increased stratification, resulting in a shallower mixed layer with increased light, has been postulated to favor diatom growth, but at the same time decreases in upwelling also due to stratification have been predicted to more negatively impact large diatoms compared to small phytoplankton [142]. Meanwhile a region around the Antarctic Peninsula has seen overall summer phytoplankton abundance decrease by 12% over the past 30 years [143]. Warming temperatures may also alter adaptation to other polar conditions, such as darkness [144]. 

Microalgae play a key role in biogeochemical cycling. Species composition, abundance, cell size and life history all determine the drawdown of organic matter and C, N, and P are sequestered in different ratios depending on such factors [145]. Furthermore, changes in lipid composition and other metabolic shifts associated with adaptations of polar microalgae to altered niches [32,146] will have complex effects on food quality, community structure and biogeochemical processes [147]. Importantly, polar microalgae are key players in two major climate feedback loops that mitigate global warming trends: deep ocean carbon sequestration [27] and cloud condensation sulfate particles [148]. Changes in species composition and/or environmental variables significantly affect the fluxes within these feedback loops but to what extant is still largely uncertain. Clearly, the need to better understand feedbacks between climate, primary production, and biogeochemical loops in polar regions is paramount, yet models are ripe with uncertainties inherent in attempts to define function based on broad taxonomic classifications, such as nano *versus* picoplankton or diatom *versus* haptophyte [149]. “Omics” techniques continue to generate a wealth of data towards understanding acclimation potentials and metabolic fluxes, as well as elucidating niche separation and climate change adaptability. The next major hurdle will be advancing our ability to quantify and model different molecular/metabolic strategies to give a finer resolution on functional groups and biogeochemical fluxes, particularly in the face of new ecological pressures. This requires close collaborations between molecular biologists and modelers in order to develop a holistic approach based on genomic and biochemical data. Such an integrative systems ecology approach will provide mechanistic insights into how climate change will impact polar phytoplankton communities.

## 5. Conclusions

Only over the past decade have modern molecular genomic, transcriptomic, proteomic, and metabolomic tools been applied to polar microalgae. Although still in its infancy, great insights have already been made in regards to adaptation and acclimation mechanisms of polar microalgae using these new techniques. As we improve our understanding of polar bioenergetics, resource management, metabolic fluxes, and community composition, our ability to understand feedbacks of polar microalgae on global biogeochemical processes will become clearer. Furthermore, discovery of novel genes and pathways could have profound impacts on biotechnological applications.
